# The influence of various welding wires on microstructure, and mechanical characteristics of AA7075 Al-alloy welded by TIG process

**DOI:** 10.1038/s41598-024-69227-4

**Published:** 2024-08-16

**Authors:** Ramy A. Fouad, Mohamed I. A. Habba, Yousef G. Y. Elshaghoul, Mohamed M. El-Sayed Seleman, Khalid M. Hafez, F. S. Hamid, Waheed S. Barakat

**Affiliations:** 1 Mechanical Department, Faculty of Technology and Education, Suez University, Suez, 43221 Egypt; 2 Mechanical Engineering Department, Faculty of Engineering, Suez University, Suez, 43221 Egypt; 3 Metallurgical and Materials Engineering Department, Faculty of Petroleum and Mining Engineering, Suez University, Suez, 43221 Egypt; 4 Central Metallurgical Research and Development Institute (CMRDI), P.O. Box 87, Helwan, 11421 Egypt

**Keywords:** AA7075 Al-alloy, Engineering applications, Mechanical characteristics, Welding wires, Strain hardening capacity, Fracture surface morphology, Mechanical engineering, Structural materials

## Abstract

Owing to their exceptional mechanical properties, the various welding wires used to combine aluminum can meet the needs of many engineering applications that call for components with both good mechanical and lightweight capabilities. This study aims to produce high-quality welds made of AA7075 aluminum alloy using the GTAW technique and various welding wires, such as ER5356, ER4043, and ER4047. The microstructure, macrohardness, and other mechanical characteristics, such as tensile strength and impact toughness, were analyzed experimentally. To check the fracture surface of the AA7075 welded joints, the specimens were examined using optical and scanning electron microscopy (SEM). A close examination of the samples that were welded with ER5356 welding wire revealed a fine grain in the weld zone (WZ). In addition, the WZ of the ER4043 and ER4047 welded samples had a coarse grain structure. Because the hardness values of the welded samples were lower in the WZ than in the base metal (BM) and heat-affected zone (HAZ), the joints filled with ER5356 welding wire provided the highest hardness values compared to other filler metals. Additionally, the ER4047 filler metal yielded the lowest hardness in the weld zone. The welding wire of ER5356 produced the greatest results for ultimate tensile stress, yield stress, welding efficiency, and strain-hardening capacity (Hc), whereas the filler metal of ER4043 produced the highest percentage of elongation. In addition, the ER4047 fracture surface morphology revealed coarser and deeper dimples than the ER5356 fine dimples in the welded joints. Finally, the highest impact toughness was obtained at joints filled with the ER4047 filler metal, whereas the lowest impact toughness was obtained at the BM.

## Introduction

Aluminum alloys (7xxx series) are high-specific-strength, low-density materials commonly used in applications that require lightweight metals. Owing to their desirable properties, these series are used in various sectors and industries, such as aircraft parts, naval, and automobiles^1,2^. The Al–Zn–Mg Al-alloy (7075 Al-alloy) contains Zn and Mg in quantities up to 8% and 3% by weight, respectively. Both alloying elements have a high solid solubility in aluminum alloys. The addition of Mg causes a significant increase in the precipitation-hardening properties. Thus, 7075 Al alloys are used in automobile brakes, body panels, air deflector parts, brake covers, fuel tanks in spacecraft^3–5^, and seat slides of autos^6^ owing to their good mechanical characteristics (high hardness and tensile stress), machinability, hot workability, and corrosion resistance^7^. In recent years, numerous studies and applications related to the use of 7xxx alloys in various industrial sectors have increased^8–10^. Welding of similar/dissimilar metals is the basic method of joining and is extremely important for these metals in numerous complex engineering equipment industries^11^. Fusion welding is very important for joining Al 7075 alloys because of its high sensitivity to hot cracking during welding operations. In general, the welding operation of aluminum alloys is regarded to be more difficult than that of other materials such as steel owing to their high thermal and electrical conductivity^12^, tendency of the Al alloy to form aluminum oxide (Al_2_O_3_) during welding, high thermal expansion coefficient, and low stiffness. Therefore, different welding techniques such as Friction Stir Welding (FSW)^13–16^, Laser Beam Welding (LBW)^17,18^, Gas Tungsten Arc Welding (GTAW), Resistance Spot Welding (RSW), Gas Metal Arc Welding (GMAW)^19,20^, etc. have been used to weld various Al alloys. The GTAW technique is widely used in the joining process of Al alloys, and it produces good weld samples and quality. Among fusion arc welding methods, GTAW is mainly used in industries that require a high level of welding quality or precision^21,22^. This method uses a non-consumable electrode that passes protective gas through the torch to shield the electrode and molten weld pool^23^. Hence, the choice of appropriate welding parameters is very important, as filler metals are the main key to averting hot cracking. Usually, welded aluminum alloys from the 7xxx series, such as the 7075 Al alloy, mainly involve 5xxx series filler metals^24^. Abbass and Abd Ul-Qader.^25^ investigated the influence of filler metals ER4047, ER4043, and ER5356 on the Microstructure and mechanical properties of GTAW-welded AA2024-T3 similar butt joints. Their findings revealed that the highest tensile strength was obtained for joints welded using ER5356 compared with other welding wires. The most even distribution of hardness values was observed in joints filled with ER4047 and ER4043 filler metals. Also, Shi. et al.^12^ proposed the four types of filler metals (ER1100, ER5356, ER4043, and ER4047) welded by the technique of double-electrode GMAW (DE-GMAW) on mechanical characteristics of dissimilar Al/Cu joints, and their findings revealed that the hardness values ​​of the weld region were significantly improved when using the welding wire type ER4047. Additionally, ER4047 provides the highest tensile strength for weld-specimen weldments compared to other filler metals. Ishak. et al.^26^ used three different filler wires of ER5356, ER4043, and ER4047 to weld the 6061 Al-alloy and they found that the microstructure of the joints welded by the filler metal of ER5356 produced a fine grain structure compared to samples manufactured by filler metals of ER4043, and ER4047. In addition, the highest hardness values, tensile strength, and yield strength were obtained in samples made with ER5356 compared to counterpart ER4043 and ER4047 wires. Furthermore, Verma. et al.^27^ experimented with two filler metals, ER5356 and ER4043, on the welds of dissimilar Al-alloys of 5083-O and 6061-T6 made by the GTAW process. They observed that tensile strength and hardness values for welds made using ER5356 filler metals can increase tensile strength and hardness values by 28.5% and 67.9%, respectively, compared to ER4043. This current work aims to produce high-quality welds made of AA7075 aluminum alloy using the GTAW technique and various welding wires, such as ER5356, ER4043, and ER4047. The selection of filler metals ER5356 (Al–Mg), ER4047 (Al–Si), and ER4043 (Al–Si) for this study was based on several key factors. ER5356, which is rich in Mg, was chosen for its potential to enhance the strength and corrosion resistance of the weld. ER4047 and ER4043, both silicon-rich alloys, were selected for their ability to reduce hot cracking susceptibility, which is a common issue in welding 7xxx series aluminum alloys. ER4047, with a higher silicon content (12% Si) than ER4043 (5% Si), allows for a comparison of the effects of silicon concentration. These filler metals represent distinct alloy systems (Al–Mg and Al–Si), enabling a comprehensive evaluation of their impact on the microstructure and mechanical properties of AA7075 welds. By comparing these three filler metals, the microstructure, hardness, and other mechanical characteristics, such as tensile strength and impact toughness, were analyzed experimentally. Unlike previous studies, this work provides a comprehensive comparison of these specific filler metals on AA7075 alloy, offering valuable insights into their effects on weld quality and mechanical properties. By focusing on this particular combination of base material and filler metals, the current work addresses a gap in the existing literature and provides more information on the effect of applied welding wires on the properties of AA7075 aluminum alloy welded by the GTAW technique.

## Materials and methodology of research

### Materials

**Figure 1:**
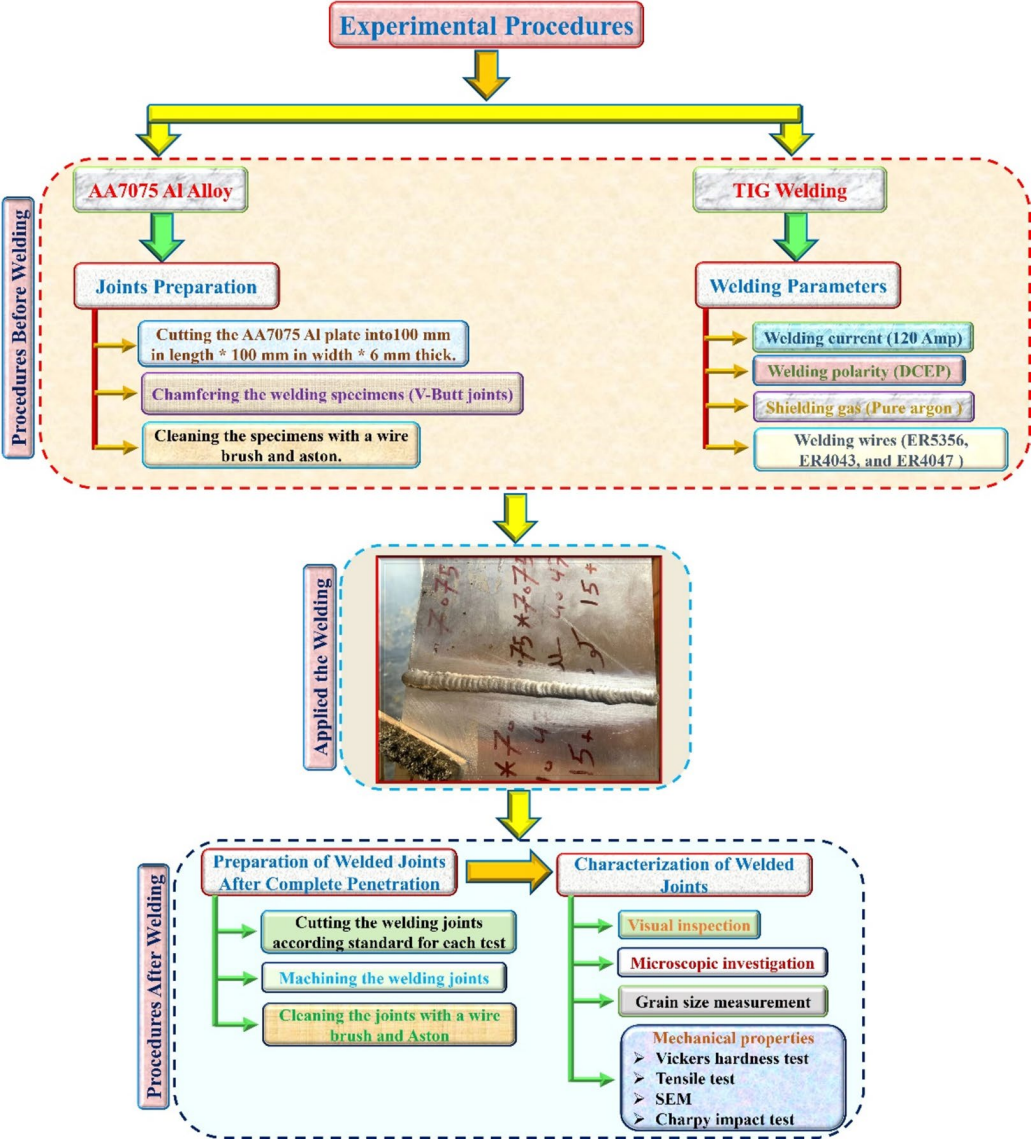
Presents the applied experimental procedures.

This study concentrates on a commercial Al–Zn–Mg aluminum alloy, specifically with a thickness of 6 mm, provided by the Egyptian Aluminum Company, Egypt. It involves the use of distinct welding wires made from aluminum alloys of grades ER5356 (Al–Mg), ER4047 (Al–Si), and ER4043 (Al–Si) provided by the ESAB Company, Cairo, Egypt, each with a diameter of 3.2 mm. The composition and mechanical properties of the base material and filler metals (according to the datasheet provided by the ESAB Company) are detailed in Tables 1 and 2, respectively. The experimental plan for this study is illustrated in Fig. 1.

**Table 1:** The composition for Al–Zn–Mg alloy and welding wires, wt%.

Element	Mg	Zn	Fe	Cu	Si	Cr	Ti	Mn	Al
Al–Zn–Mg alloy	2.35	5.46	0.38	1.44	0.13	0.24	0.03	0.11	Bal
ER5356	4.9	0.01	0.15	0.015	0.025	0.11	0.09	0.14	Bal
ER4047	0.10	0.10	0.8	0.054	13.0	–	–	0.017	Bal
ER4043	0.05	0.1	0.8	0.025	5.3	0.01	0.02	< 0.05	Bal

**Table 2:** The mechanical properties for Al–Zn–Mg alloy and welding wires.

Element	Ultimate tensile stress, MPa	Yield stress, MPa	Elongation, %
Al–Zn–Mg alloy	572	503	11
ER5356	275	126	18
ER4047	130	60	5
ER4043	120	40	8

### Welding process

**Figure 2:**
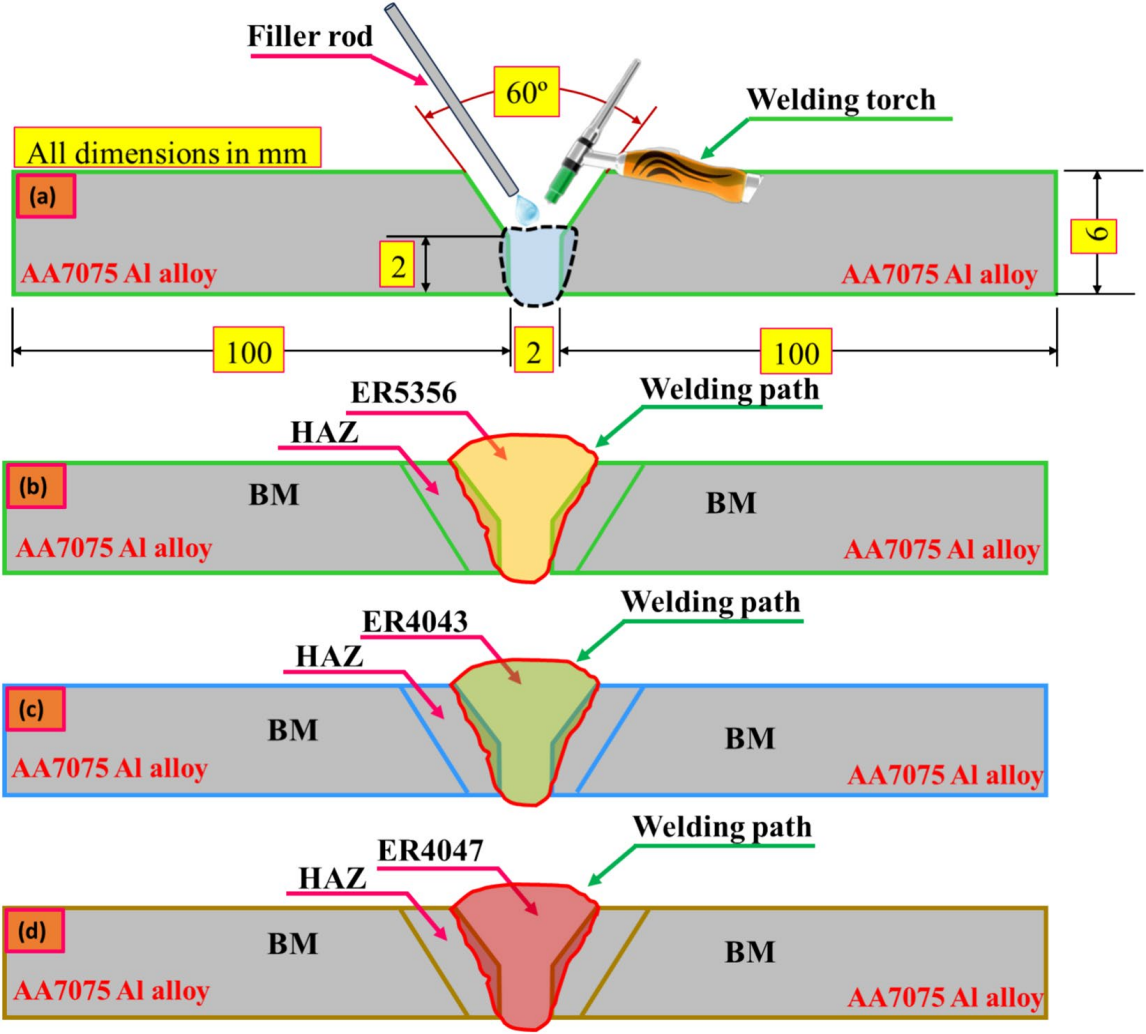
Schematic drawing of the AA7075 welded joints using different filler rods.

Using Wire-cut Electrical Discharge Machining (WEDM), AA7075 BM was prepared for GTAW in a flat position with dimensions of 150 mm in length, 100 mm in width, and 6 mm in thickness. To create a single V-groove with a 60° angle, 2 mm root gap, and 2 mm root face, each sample was milled to a 30° angle using a milling machine, as shown in Fig. 2. Prior to welding, the BM was cleaned with acetone and a wire brush to remove any inclusions, grease, grime, and oxides. Butt welds were created using a fixed current and voltage in the GTAW process with DCEN polarity. Pure argon gas (99.99%) at a flow rate of 10 L/min was used to protect the welding pool. In this work, a 2.4 mm diameter non-consumable AWS EWTH-2 tungsten electrode was utilized.

### Microstructure examination for welded joints

**Figure 3:**
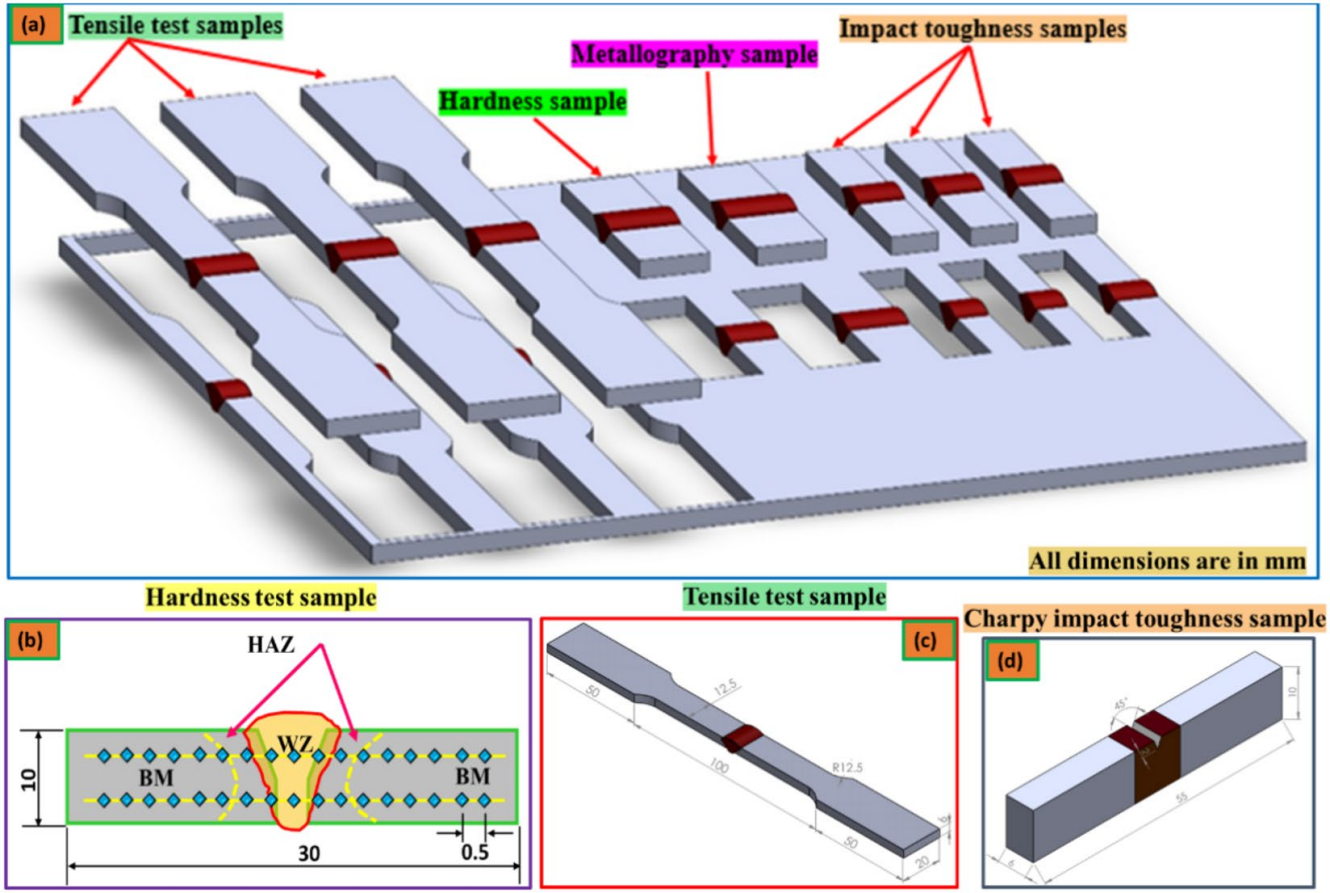
Sketch of the testing samples; (**a**) mechanical and metallographic examination samples, (**b**) hardness specimen, (**c**) tensile specimen, and (**d**) impact test specimen.

The fabricated similar butt welds were examined for the microstructures of different welding regions. Metallographic samples were prepared by cutting the transverse section across the weld line, as shown in Fig. 3a. The microstructure samples were ground using different degrees of emery papers (800, 1000, 1200, 1500, and 2400 grit), followed by polishing with a 0.3 μm Al_2_O_3_ paste. Microscopic investigations of Al–Zn–Mg alloy butt welds were carried out by etching with a ified Keller’s reagent (2 mL of HF, 3 mL of HCl, 5 mL of HNO_3,_ and 90 mL of H_2_O). The microstructure of the different zones of interest, such as the WZ, HAZ, and BM combination, was viewed and captured with an optical microscope (OM, Nikon Instruments Inc., Tokyo, Japan) coupled with the analysis of these images using software. The grain sizes for all welding joints produced were measured from photomicrographs obtained in the polarized light of a light microscope (LM) using the random secant method.

### Mechanical characterization for welded joints

To analyze the mechanical behavior of all welding specimens, the tensile properties, including UTS, YS, and El%, as well as the impact test, were carried out at room temperature on a universal testing machine (WDW-300D, Guangdong, China). A tensile test conducted in accordance with ASTM E8M-04 was utilized to ascertain the mechanical behavior of the welded alloy AA7075; see Fig. 3c. On the polished cross-sections, the macrohardness profiles were also measured using a Vickers hardness tester machine (HWDV-75, TTS Unlimited, Osaka, Japan) with a 0.5 kg load and a 15 s dwell time at all various welding locations. As per ASTM E384-09, hardness test samples were utilized for every welding zone; Fig. 3b illustrates this. Finally, a JB-300B Charpy Pendulum Impact Testing Machine was used to measure the impact energy at room temperature. As shown in Fig. 3d, the impact test samples were cut and prepared in accordance with ASTM E23-01.

## Results and discussion

### Visual inspection of weldments

**Figure 4:**
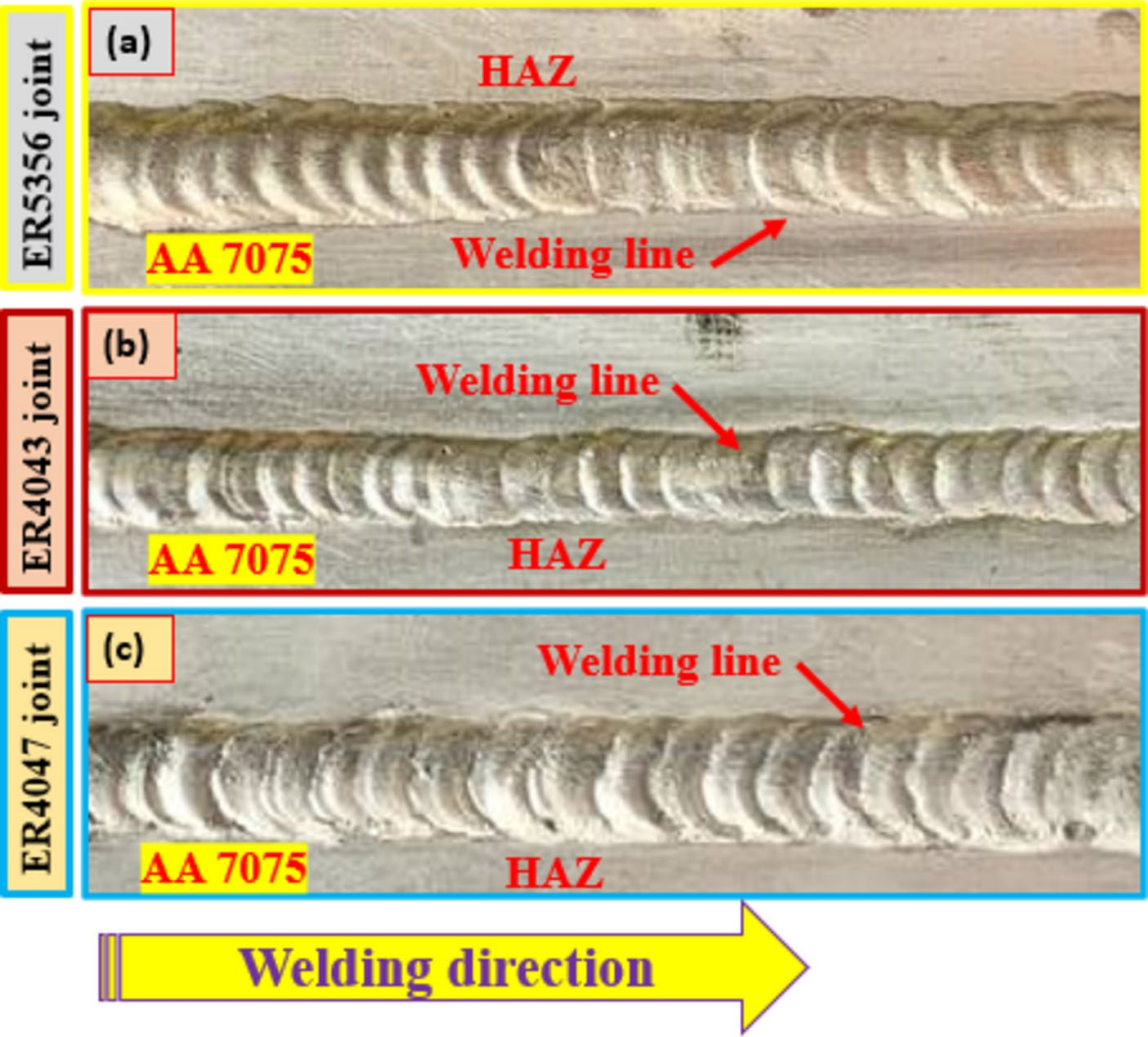
Surface appearance for welded joints; (**a**) welded sample by ER5356, (**b**) welded sample by ER4043, and (**c**) welded sample by ER4047.

Figure 4 shows the effect of various filler metals (ER5356, ER4043, and ER4047) on the quality of the weldments produced. In addition, all welding variables allowed us to produce weld joints with free defects, such as cracks and porosity. The welded joints produced by ER4043 had thin and smooth welding lines, whereas those produced by ER5356 and ER4047 welding wires had thick welding lines. This was the most noticeable difference in the visual appearance of the welded joints. Based on a visual examination of the production welding specimens, no defects appeared in the weld bead shape on both the face and root sides. Thus, it is impossible to say with absolute certainty that the welding wires used with ER5356, ER4043, and ER4047 had any effect on the occurrence and formation of welding defects. Figure 4a–c displays the morphology of the welded joints produced with various types of welding wires. Additionally, all welded joints were visually inspected to ensure that the samples were defect-free. Table 3 indicates the visual inspection report results for the AA7075 aluminum alloy joints produced using various welding wires: ER5356, ER4043, and ER4047. Based on the visual inspection results, all the welded joint surfaces were considered acceptable. However, a few points should be noted for manufacturing welded joints such as face joint reinforcements, which vary in 1.28–1.56 mm thickness, as presented in Table 3. According to the positive visual examination results, the selected welding wires are perfect for joining AA 7075 aluminum alloys using TIG welding.

**Table 3:** The visual examination report of TIG butt joints produced of aluminum alloy AA7075 that were welded using various welding wires.

Samples no	Welding technique	Sample dimensions, mm	Evaluation	Statements
ER5356	TIG	200 × 200 × 6	Approved both sides	The welding path width is 9 mm Face reinforcement is about 1.43 mm Plate distortion is not recommended
ER4043		200 × 200 × 6		The welding path width is 8.42 mm Face reinforcement is about 1.28 mm
ER4047		200 × 200 × 6		The welding path width is 9.81 mm Face reinforcement is about 1.56 mm

### Microstructure of weldments

**Figure 5:**
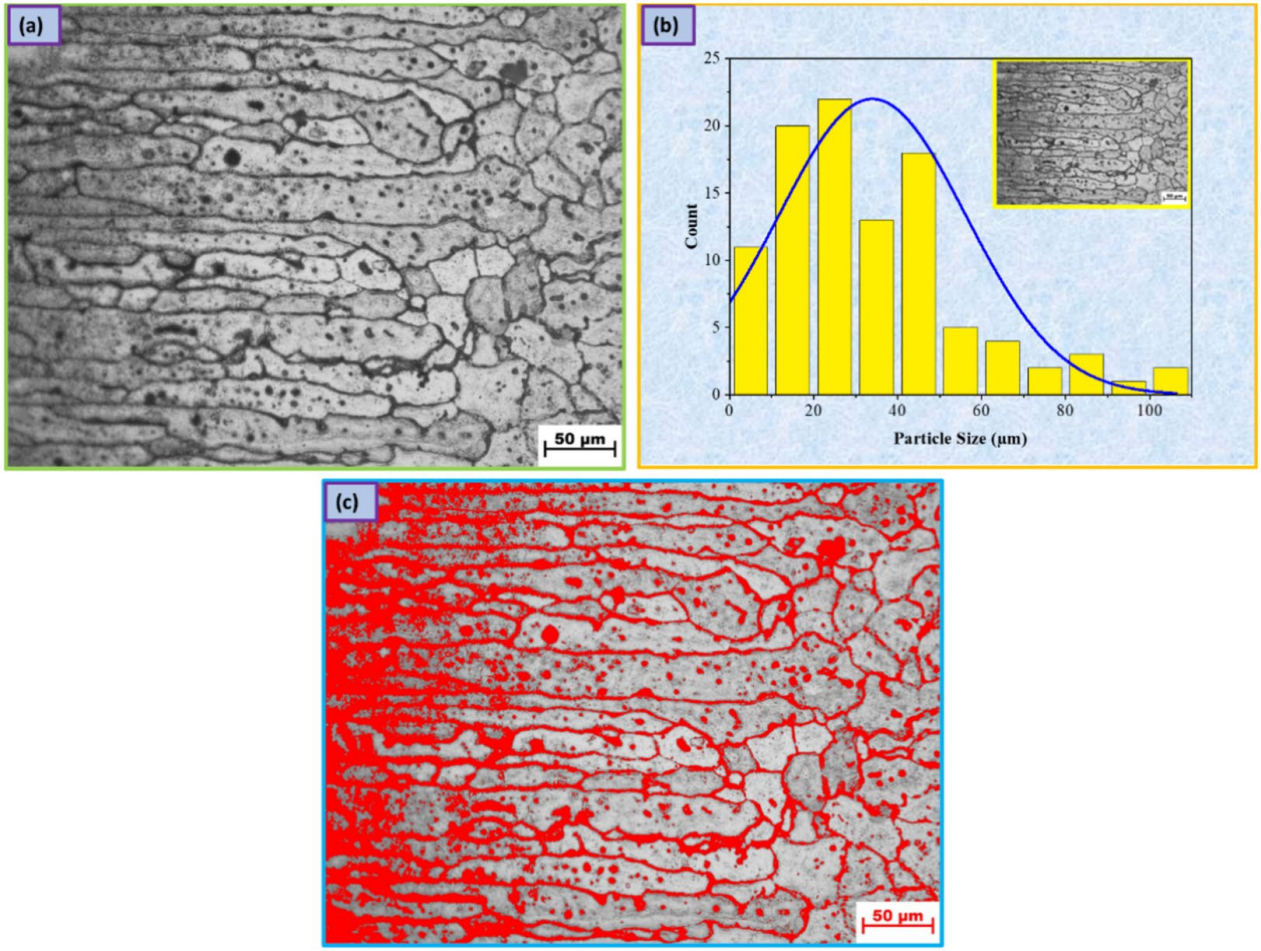
Microstructure images for initial material AA7075 Al alloy.

Figure 5a illustrates the microstructure of AA7075's initial material, which represents elongated grains along the rolling direction, accompanied by light-gray FeAl_3_ particles and spheroidal MgZn_2_ particles (black precipitates) in the aluminum solid solution^28^. Hence, the aluminum alloy of the 7XXX series was significantly influenced by its secondary phases. In addition, numerous studies have thoroughly investigated and detailed the nature of typical secondary phases^29–31^. The 7XXX family of aluminum alloys primarily contains four types of intermetallic compounds as significant secondary phases: η-phase (MgZn_2_), S-phase (Al_2_CuMg), T-phase (Al_2_Mg_3_Zn_3_), and θ-phase (Al_2_Cu). Insoluble primary Fe-rich phases (Al_7_Cu_2_Fe, Al_3_Fe, and α-AlFeSi), Si-rich phases (Mg_2_Si and SiO_2_), and Mn-rich phases (Al_6_(Fe, Mn), Al_5_Si_2_(Fe, Mn), and Al_3_(Fe Mn Cr)) would also form in the alloys owing to the presence of Mn, Fe, and Si^32^. Additionally, the density, size, shape, and distribution of the intermetallic phases that are frequent in AA7075 Al alloys have a significant effect on the mechanical behavior, as well as the alloys' tendency toward solidification cracking^32–34^. In addition, the initial material average grain size was measured and analyzed along the rolling direction by the ImageJ software program and the linear intercept method, which was found to be 33.95 ± 9 µm, as shown in Fig. 5b. In addition, the volume fraction of the η-phase (MgZn_2_) was measured using the ImageJ software program, and was found to be 3.86%, as shown in Fig. 5c. Therefore, the solidification cracking susceptibility of AA7075 basic material can be reduced to acceptable levels by modifying it with excess silicon (using the 4xxx series Al–Si filler alloys) or excess magnesium (using the 5xxx series Al–Mg filler alloys)^35,36^. To inhibit the propagation of hot cracks during and after the welding process, a 4XXX filler metal wire containing Si is typically utilized. In other situations, the strength of the welded sample is enhanced by using the 5XXX filler metal^37^.

**Figure 6:**
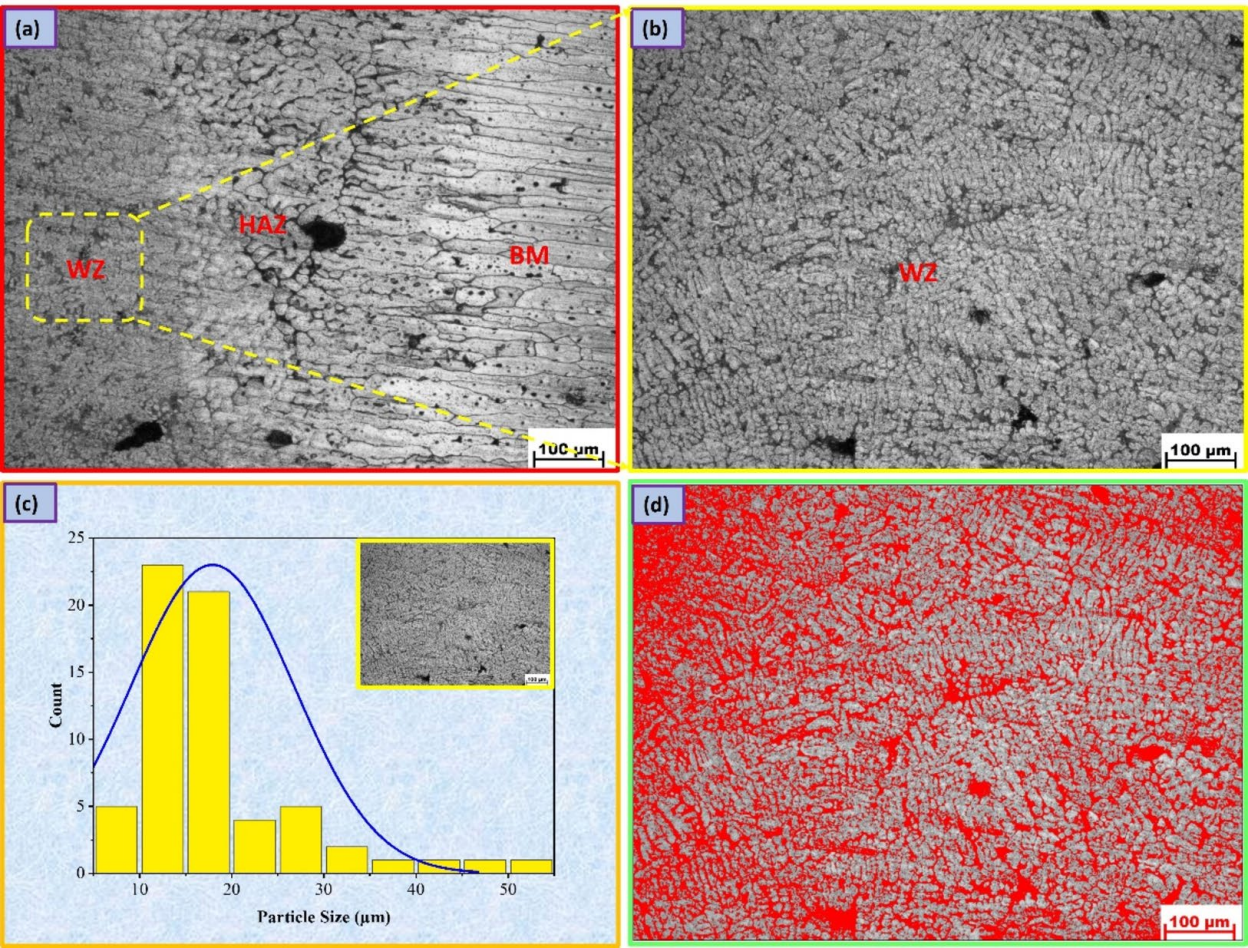
Microstructure images for welded joints by ER5356; (**a**) transition regions, (**b**) WZ, (**c**) grain analysis for WZ, (**d**) and volume fraction of MgZn_2_ phase.

**Figure 7:**
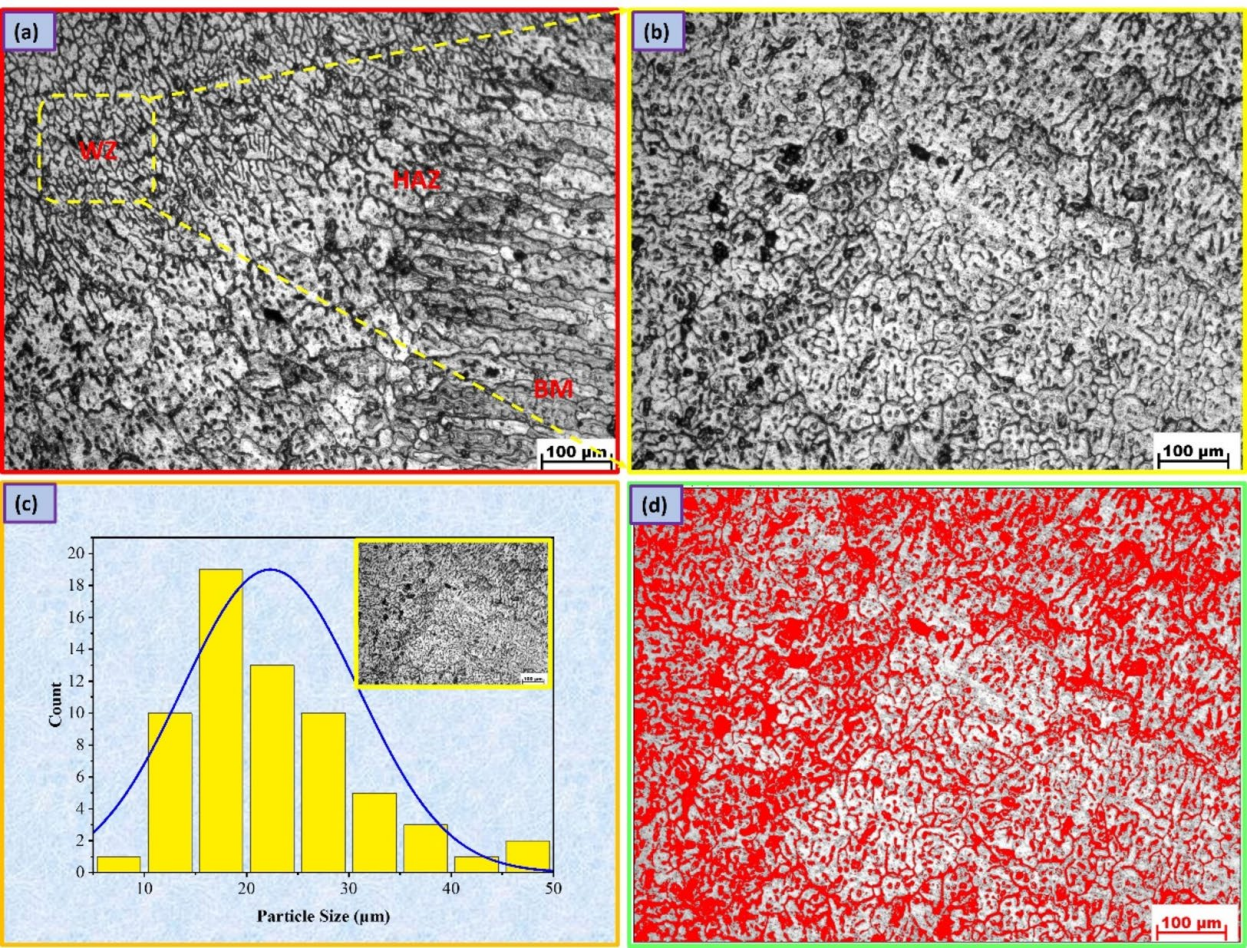
Microstructure images for welded joints by ER4043; (**a**) transition regions, (**b**) WZ, (**c**) grain analysis for WZ, (**d**) and volume fraction of MgZn_2_ phase.

**Figure 8:**
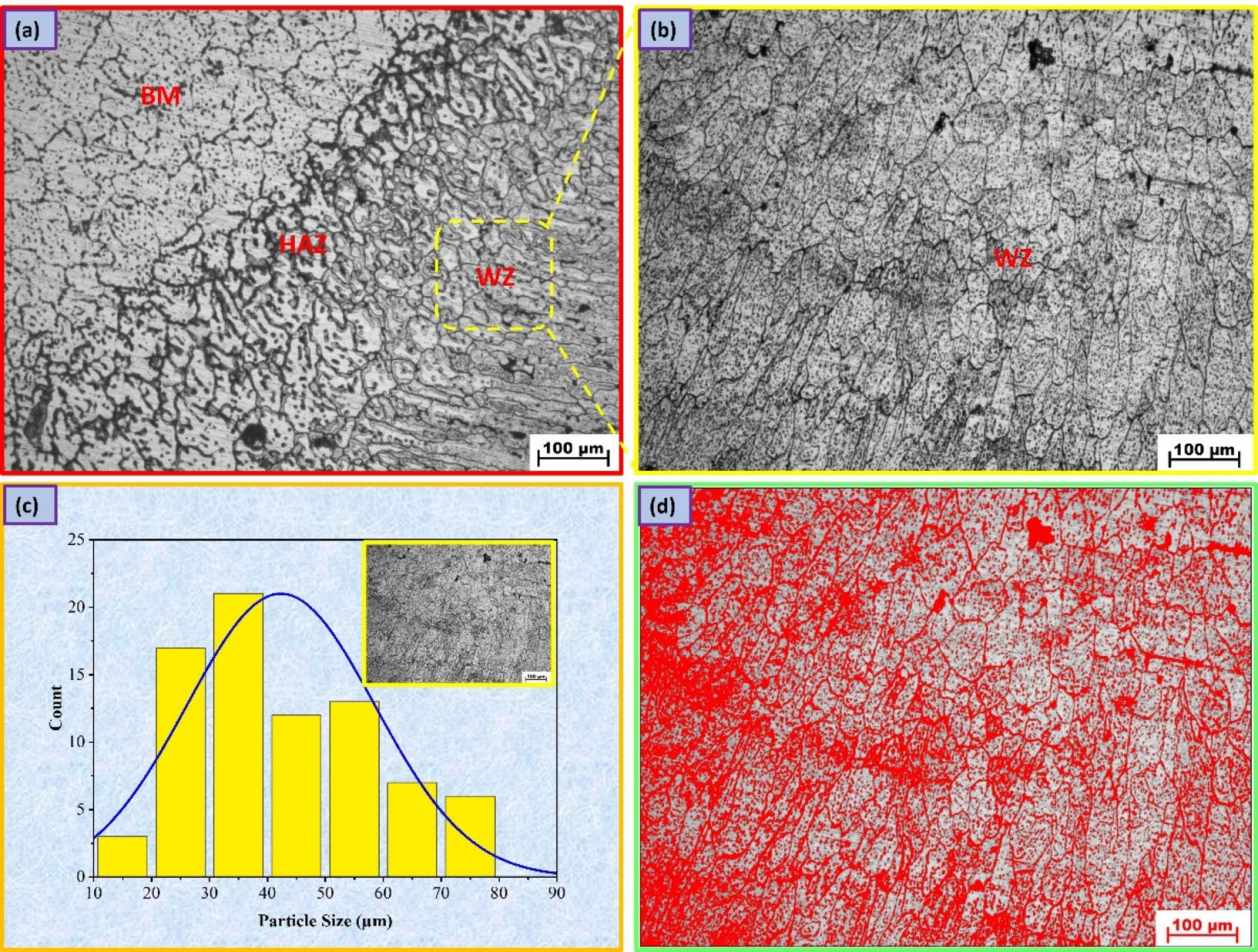
Microstructure images for welded joints by ER4047; (**a**) transition regions, (**b**) WZ, (**c**) grain analysis for WZ, (**d**) and volume fraction of MgZn_2_ phase.

Figures 6, 7, 8 display the optical microscopy images for various welding regions of the AA7075 Al alloy welded by TIG welding with different welding wires. Additionally, the BM long, flat micrograins are oriented in the direction of the rolling mill as a result of deformation, and when welding occurs, the impact of fusion and heat input causes the grains to become coaxial and larger in size. This phenomenon is clearly observed in the transition regions of the welding lines, as shown in Figs. 6a, 7a, and 8a^38^. However, in the HAZ, as shown in Figs. 6a, 7a, and 8a, the microstructure is mainly influenced by two types of solid-state reactions^39^: (a) the dissolution of precipitates and the growth of grains in areas that experience higher peak temperatures and (b) the partial dissolution of precipitates and the transformation from a metastable phase to a stable phase in regions that experience lower peak temperatures. The AA7075 Al alloy has a wide freezing range, resulting in the presence of coarse columnar grains and partial melting at the grain boundaries in the region between the WZ and HAZ.

A fine grain structure of the welded joint filled with ER5356 may be seen in the WZ, as seen in Fig. 6b,c, with a grain size of 17.91 ± 4 µm, an area volume fraction of MgZn_2_ of 5.83%, and an average grain size of the MgZn_2_ phase of 3.43 µm^40^, as seen in Fig. 6d. Fast cooling rates in the related weld are attributed to the generally fine weld zone microstructure and chemical composition of ER5356 filler metals. In addition, the WZ, where melting and resolidification occur after welding, as well as a zone damaged by the heat of the welding operation, must be considered. The main factors influencing the creation of the weld metal in the weld zone are diffusion, temperature gradient, and solidification growth rate. The formation of a eutectic constituent at the final stage of solidification, which removes the majority of the materials required for precipitation reactions from the matrix, limits precipitation aging^41^. On the other hand, the grain size of the welded joint filled with ER4043 filler metal is 22.33 ± 7 µm (Fig. 7b,c) with an area volume fraction of MgZn_2_ of 4.77%, and the grain size of MgZn_2_ is 4.77 µm, as seen in Fig. 7d, while the grain size for the welded joint filled with ER4047 filler metal is 42.31 ± 11 µm (Fig. 8b,c) with an area volume fraction of MgZn_2_ of 8.76% and grain size of MgZn_2_ phase of 7 µm, as seen in Fig. 8d.

### Mechanical properties of weldments

**Figure 9:**
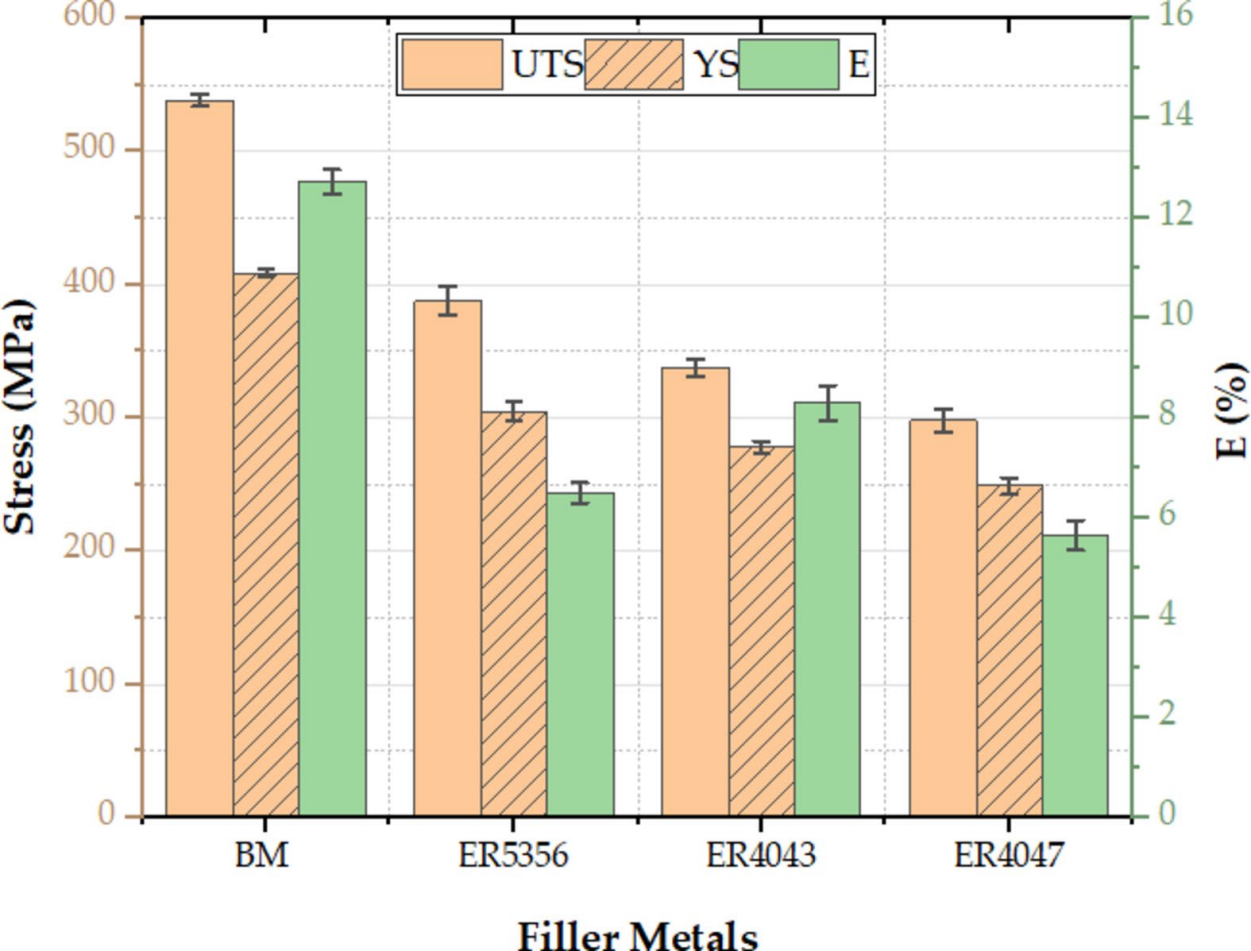
Mechanical behavior for welded joints by various welding wires.

**Figure 10:**
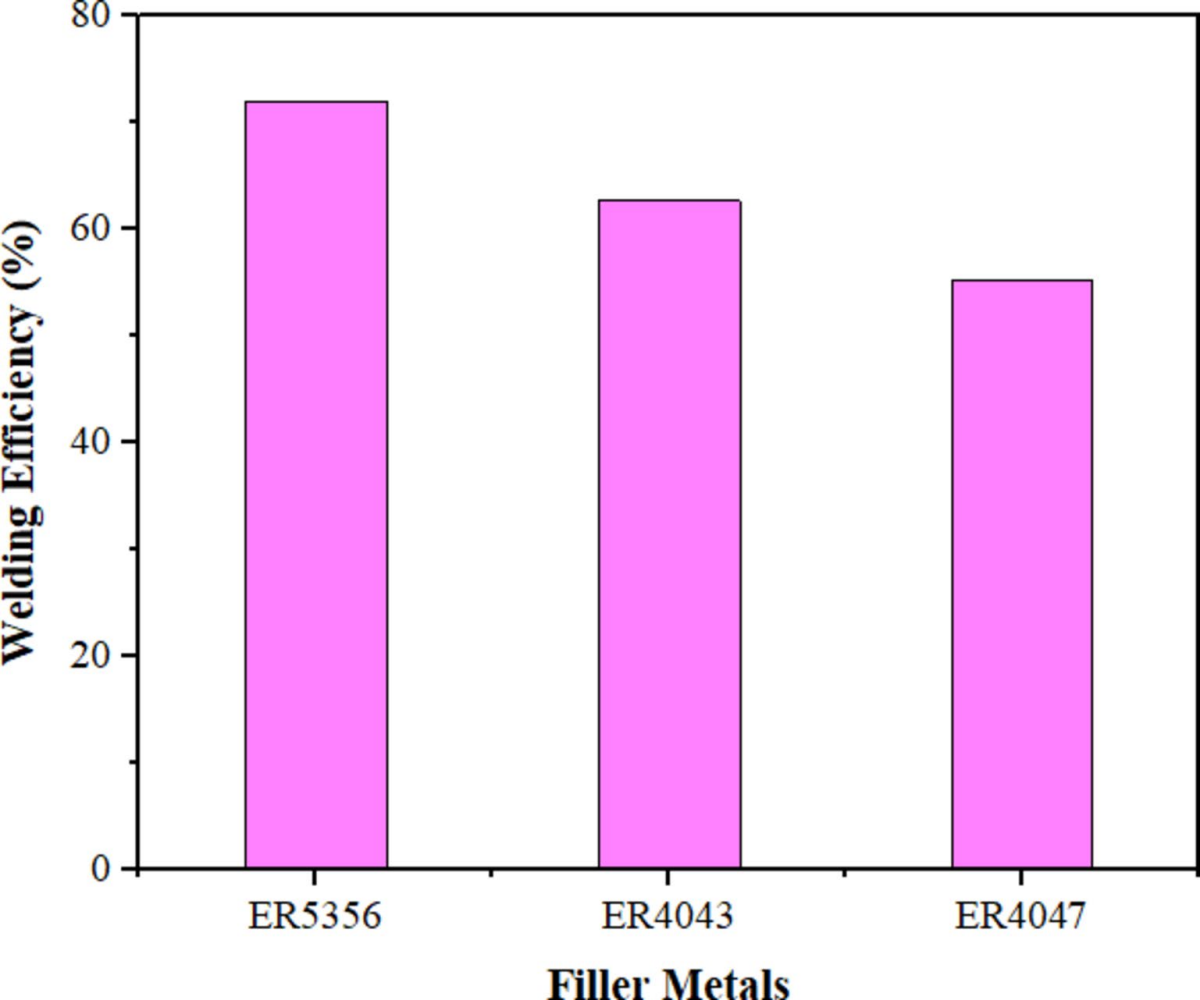
Welding efficiency for welded joints by various welding wires.

Figure 9 compares the experimental results for the mechanical behavior of the BM and welded specimens as a function of different welding wires (ER5356, ER4043, and ER4047). The welded specimens with welding wires of ER5356 have superior ultimate tensile stress (UTS), yield stress (YS), and percentage of elongation (El%) of 387.8 ± 11 MPa, 305 ± 7.4 MPa, and 6.5 ± 0.2% with an efficiency of 72% (Fig. 10) as compared to the one fabricated with rod ER4043 having UTS, YS, and El% of 337.53 ± 6.5 MPa, 278 ± 4.6 MPa, and 8.3 ± 0.35% with an efficiency of 62.7% (Fig. 10) and ER4047 having UTS, YS, and El% of 297.94 ± 8.3 MPa, 249 ± 6.2 MPa, and 5.65 ± 0.3% with an efficiency of 55.3% (Fig. 10), respectively. This is due to the difference in the composition and solidification microstructure of the filler metals. Additionally, the weld zone grain size of the sample filled with ER5356 filler metal was smaller than that of the samples filled with ER4043 and ER4047 filler metals, as indicated by^27,42^. The addition of rod ER5356 in the welded sample results in a more condensed pressure, which in turn enhances the bond strength in contrast to using filler metals ER4043 and ER4047. On the other hand, the use of fillers ER4047 and ER4043 in the samples caused a significant increase in grain size, leading to a decrease in the mechanical properties^42^. According to the Hall–Petch equation, tensile stress is inversely related to grain size^43^. The ductility of the welded samples using ER4043 filler metal was roughly 27.7% and 46.9% higher than that of the welded joints using ER5356 and ER4047 fillers, respectively. The reformed eutectic phase plays a crucial role in controlling the ductility. Fracturing started in the plastic region of the stress–strain curves for all the samples and finally occurred in the WZ. Meanwhile, the UTS-to-YS ratio can be used to calculate a material's strain-hardening capacity (Hc)^44,45^. Hc was modified to a normalized parameter by Afrin et al.^45^

1 $${\text{Hc }} = \frac{{\sigma_{{{\text{UTS }} - }} \sigma_{{{\text{YS}}}} }}{{\sigma_{{{\text{YS}}}} }} = \frac{{\sigma_{{{\text{UTS}}}} }}{{\sigma_{{{\text{YS}}}} }} - 1$$

**Figure 11:**
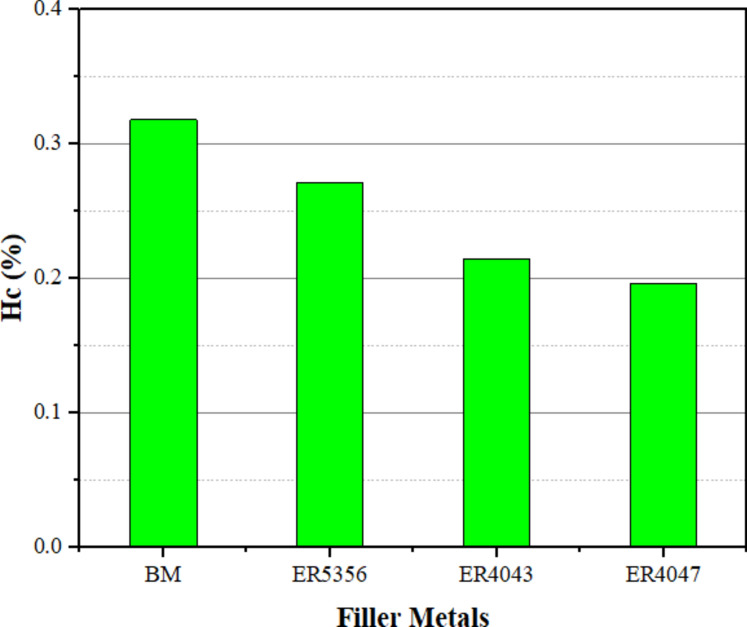
Strain-hardening capacity for welded joints by various welding wires.

**Figure 12:**
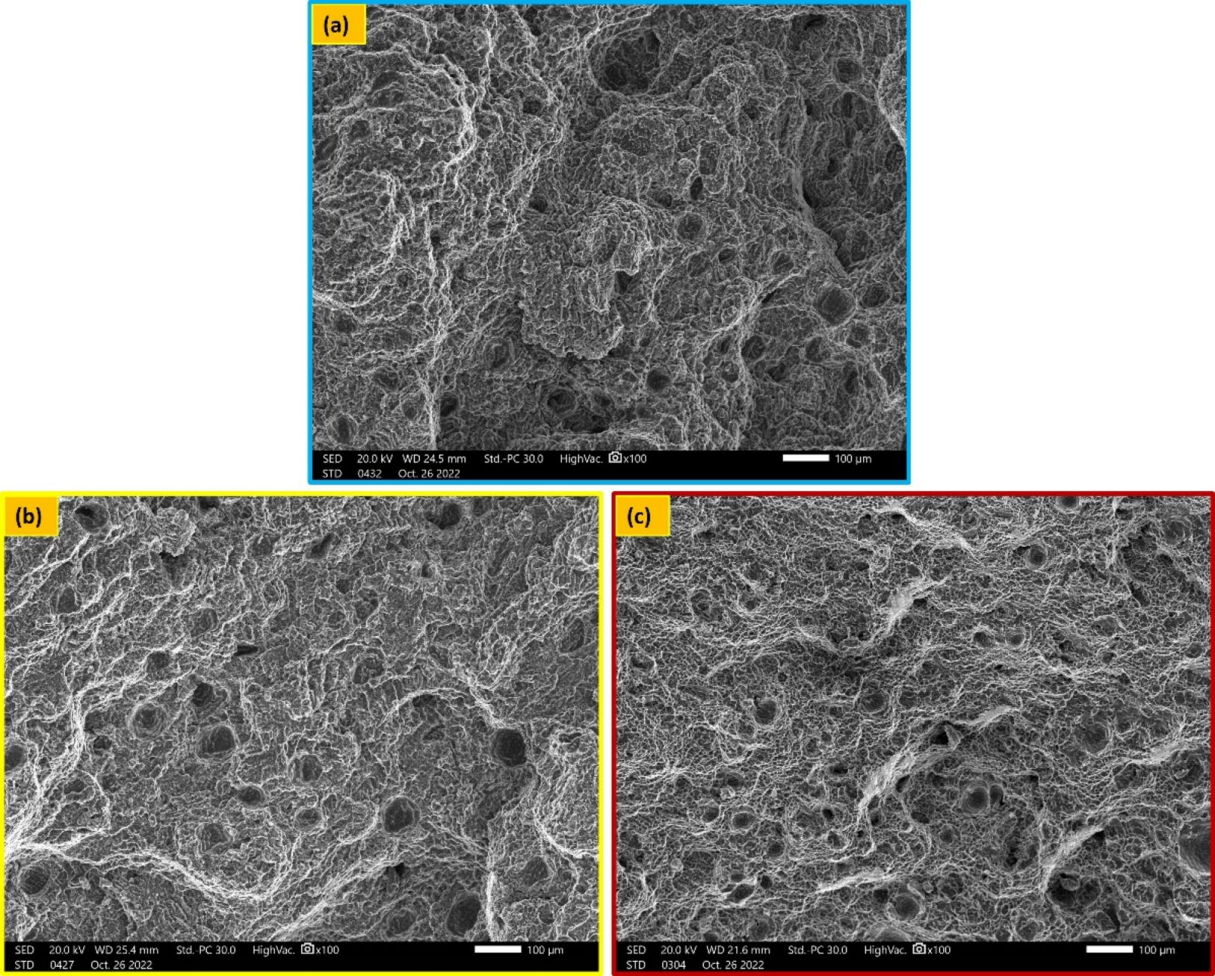
Fracture surface photos for welded joints; (**a**) BM, (**b**) welded joints by ER5356, and (**c**) welded joints by ER4047.

The material's ability to resist deformation under tension, known as the tensile hardening capacity, is somewhat dependent on its strength, specifically its Hc value, which is directly correlated with the material's strength^46^. The hardening capacity of metals is principally determined by the interaction between dislocations rather than relying on the grain size for grain boundary strengthening^47^. Hence, Fig. 11 displays the Hc of the joints that were welded using various welding wires. It was discovered that the base metal had the highest Hc value of 0.32, whereas welded joints utilizing ER4047 fillers had the lowest Hc value of 0.2. Figure 12 displays the fractography of the weld specimens obtained by scanning electron microscopy at high magnifications. Dimples, tear ridges, ductile dimples, and cleavage facets formed the fractured surface caused by the base metal and welded joints of the ER4047 welding wire, as shown in Fig. 12a,c. Because ER4047's shattered surface of the base metal and welded joints are dominated by transgranular ductile dimples. In contrast to ER5356, which has a fragmented surface with fine dimples, a dominant cup, and a cone structure, as shown in Fig. 12b. A small percentage of ripping ridges indicates that the weld may have torn relatively early in its development, when it may have been one of the primary causes of failure. However, ductile ripping dominated the failure mechanism as the tensile load increased.

### Influence of different welding wires on the macrohardness of welded samples

**Figure 13:**
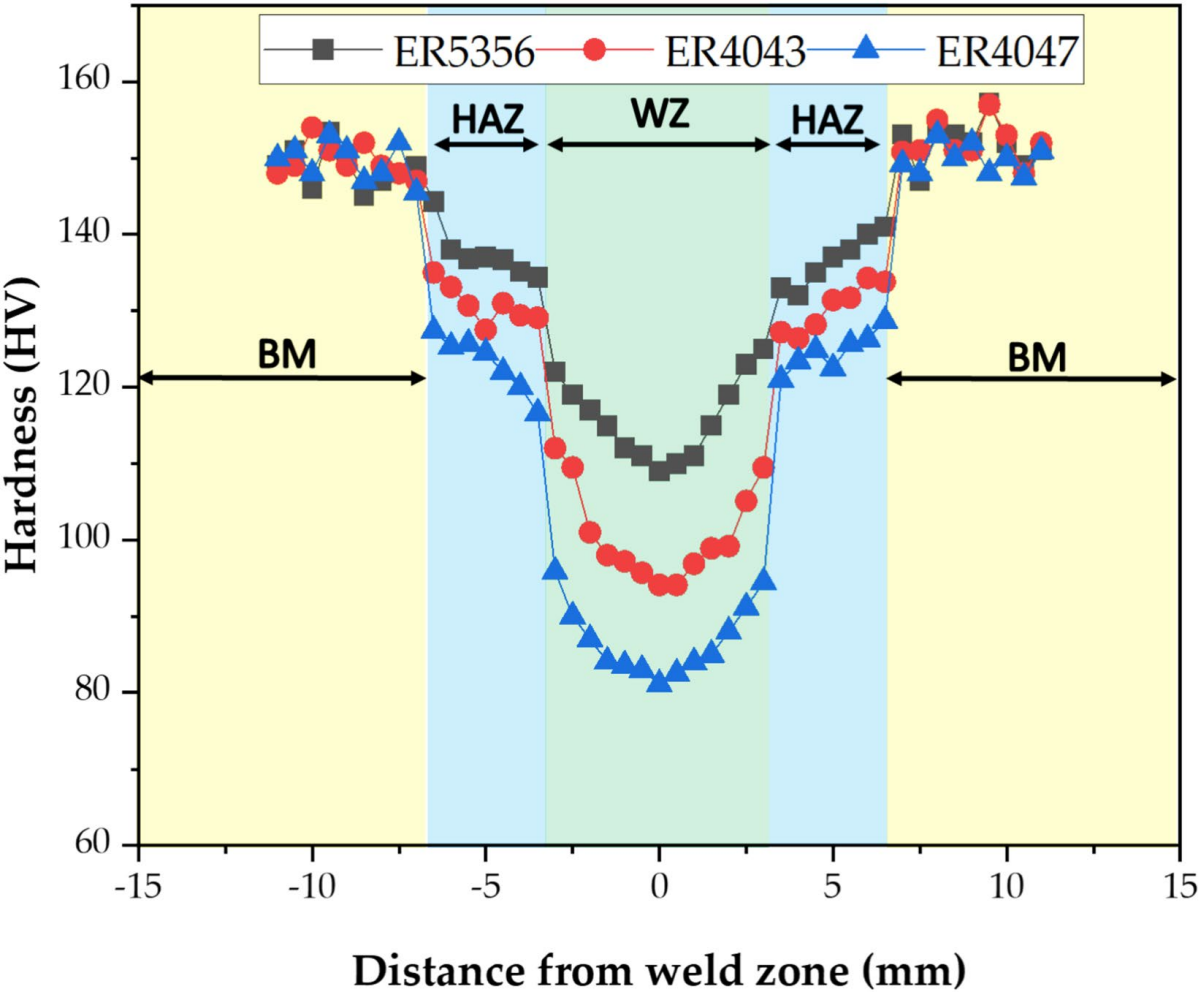
Vickers macrohardness for welded joints by various welding wires.

Figure 13 illustrates the macrohardness variations in the different welding zones from the BM, HAZ, and WZ for the AA 7075 welded samples using various welding wires from ER5356, ER4047, and ER4043. All the welding wires exhibited a similar trend in the macrohardness profile for all the produced welds. In addition, different transition zones of the weldment show a stark variation in hardness values in the WZ, HAZ, and BM, as shown in Fig. 13. The variations in macrohardness values are caused by differences in the chemical composition, morphology, and microstructure distribution of various welding wires. Additionally, the use of welding wires with different chemical compositions is mostly to blame for the noticeable decrease in WZ hardness when compared to the BM and HAZ. Thus, the lower hardness is also a result of the swift solidification of the fusion zone, which reduces the amount of time available for the precipitation of strengthening components and transformation of the 7075 Al alloy's hardening ή (MgZn_2_) precipitates into lower hardening η (Mg_2_Al_3_, Al_32_Zn_49_, and Al_2_Cu) precipitates due to the dissolution of Guinier–Preston (GP) zones in the fusion region that underwent localized heating and melting.^48–51^. It is clear that the hardness values of the WZ for all welds produced using different welding wires of ER5356, ER4043, and ER4047 are relatively changed compared to the BM and HAZ. Consequentially, the hardness values in the WZ filled by ER5356 are higher than those in the WZ welded by ER4043 and ER4047, which is primarily because the former's microstructure is dominated by a coarse grain structure.

The difference in the hardness values can be attributed to the distinct groups of elements in the filler rods. The 5xxx series (ER5356) from the Al–Mg group primarily contains magnesium as the major alloying element, whereas the 4xxx series (ER4043 and ER4047) from the Al–Si group predominantly contains silicon, which has a lower hardness. The test results indicate that the 7xxx series from the Al–Mg–Zn group exhibits higher hardness values. It was observed that the hardness values in the AA7075 BM and HAZ were greater than those in the WZ for all the welded samples. This reduction in hardness can be attributed to the decreased number of dislocations and exposure to high temperatures in this region, which reduces the hardening effect of the strengthening solutions, as supported by the literature^50,52–54^.

The formation of an unmixed zone and the migration of alloying elements from the weld and AA 7075 base material contribute to a sudden increase in hardness in the HAZ. The AA 7075 side of the HAZ had a chemical composition similar to that of the base and weld material. The slight variation in hardness in this region is mainly due to grain coarsening resulting from the different temperatures in the HAZ. The high hardness of the HAZ on the AA 7075 side makes it more susceptible to failure. Compared to the ER4043 and ER4047 filler welds, the unmixed zone of the ER5356 filler was relatively small, indicating its superiority in this regard. The limited width of the high-hardness transition zone in the ER5356 filler did not negatively impact the overall performance of the weld. Consequently, the overall structural integrity of the weld remained intact.

### Influence of different welding wires on the toughness of welded samples

**Figure 14:**
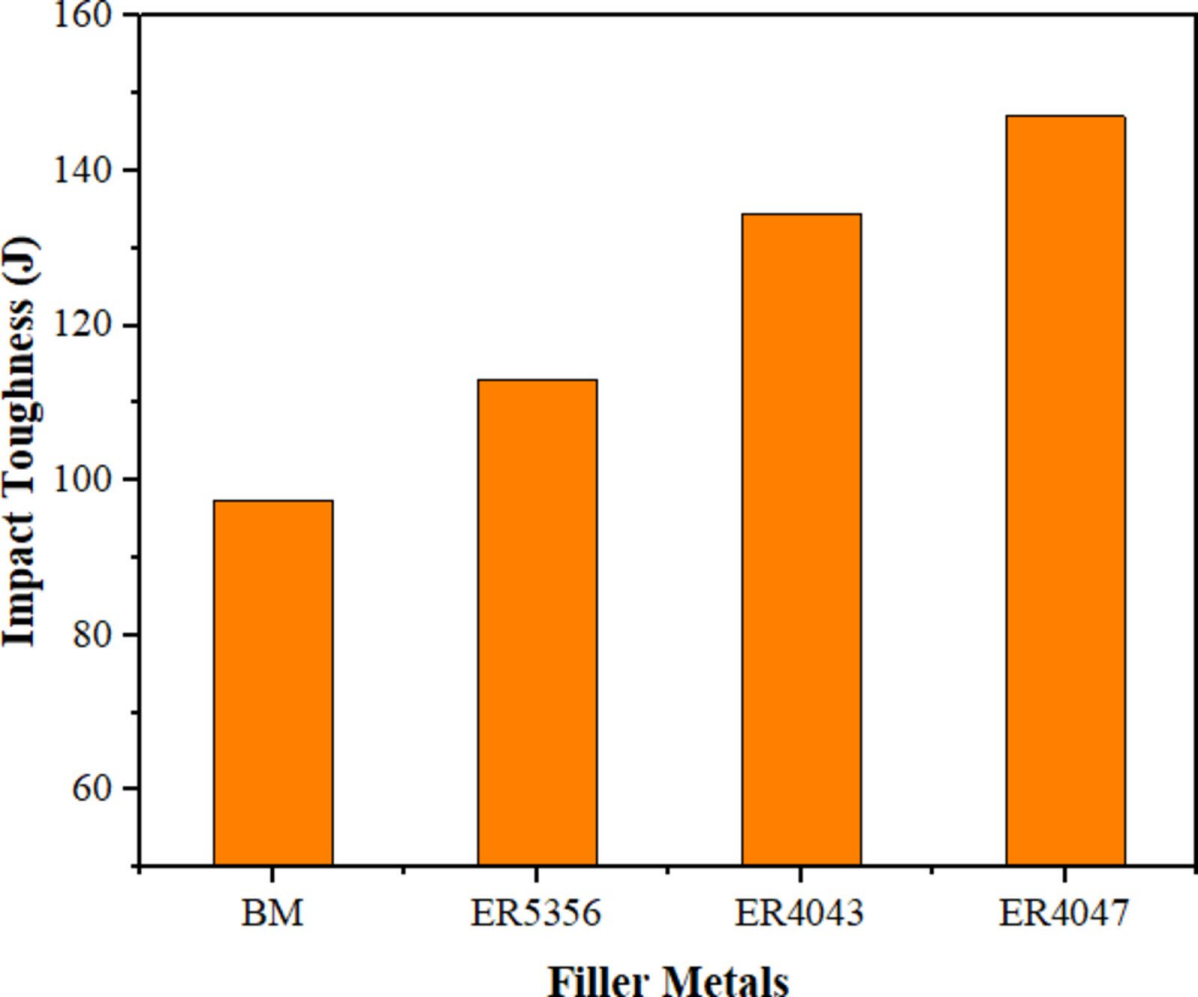
Impact toughness for welded joints by various welding wires.

Figure 14 illustrates the influence of various filler metals on the impact toughness of the welded samples. As a result, the impact toughness values of all welds were higher than 100 J in every welded sample, which is a requirement for the structural integrity of welds of a comparable kind. Compared to fillers ER5356 and ER4043, filler ER4047 offers greater impact toughness. The coarse grain structure and skeleton microstructure of the eutectic in ER4047 are known to reduce the likelihood of solidification and liquefication cracking and to give the weld a significant amount of impact toughness. The weld with the ER4047 rod exhibited an approximately 33.7% increase in impact toughness in the WZ compared with the BM. Joints welded using ER5356 welding wires exhibit lower impact toughness compared to other filler metals and are hence more vulnerable to failure. The HAZ structure and weld have nearly identical elemental compositions. Furthermore, the microstructural phases exhibit some resemblance to the alterations that arise as a result of tempering, which is attributed to the thermal fluctuations experienced during welding.

### Limitations and future directions

While this research offers valuable information about the consequences of using different filler metals on the AA7075 TIG welding process, there are several limitations that must be noted. These findings are relevant only to the AA7075 alloy and the three filler metals tested (ER5356, ER4043, and ER4047) under the specific welding conditions investigated. It is important to consider that variations in welding parameters, such as heat input or travel speed, could affect the reproducibility of the results. Furthermore, the study did not investigate the stability of the observed properties under long-term aging. Also, the research was focused on particular mechanical properties and microstructural characteristics; other properties, such as corrosion resistance or fatigue behavior, were not evaluated. Future studies could explore the effects of post-weld heat treatments or the behavior of these welds under dynamic loading conditions.

## Conclusions

In this study, the impact of welding wires ER5356, ER4043, and ER4047 on the welding quality, microstructure, and mechanical behavior of TIG weldments on an AA7075 Al alloy was investigated. The following conclusions can be drawn:

1. The welded joints using ER5356 filler metal exhibited the finest grain structure in the weld zone (17.91 ± 4 μm), compared to ER4043 (22.33 ± 7 μm) and ER4047 (42.31 ± 11 μm). This fine grain structure contributed to improved mechanical properties.
2. Joints filled with ER5356 filler metal demonstrated the highest tensile strength, yield strength, welding efficiency, and strain-hardening capacity. ER4043 provided the highest elongation, whereas ER4047 resulted in the lowest overall strength.
3. All welds showed lower hardness in the weld zone than in the base metal and heat-affected zone. ER5356 filler metal resulted in the highest hardness values in the weld zone, while ER4047 produced the lowest.
4. The base metal and joints filled with ER4047 exhibited ductile fracture modes, whereas joints filled with ER5356 showed a more brittle fracture mode.
5. Joints filled with ER4047 filler metal demonstrated the highest impact toughness, showing a 33.7% increase compared to the base metal.
6. Based on the overall performance in terms of microstructure and mechanical properties, ER5356 is recommended as the optimal filler metal for the TIG welding of AA7075 aluminum alloy among the tested options.

## Data Availability

Concerning the availability of raw data, I want to inform you that this work is part of a long research study, and the raw data will not be public at the moment but are available from the corresponding author upon request.
